# Treating Cancers Using Nature’s Medicine: Significance and Challenges

**DOI:** 10.3390/biom11111698

**Published:** 2021-11-15

**Authors:** Samson Mathews Samuel, Peter Kubatka, Dietrich Büsselberg

**Affiliations:** 1 Department of Physiology and Biophysics, Weill Cornell Medicine-Qatar, Education City, Qatar Foundation, Doha 24144, Qatar; sms2016@qatar-med.cornell.edu; 2 Department of Medical Biology, Jessenius Faculty of Medicine, Comenius University in Bratislava, 03601 Martin, Slovakia; peter.kubatka@uniba.sk

There was a time when plant-derived natural formulations were the cornerstone of ancient therapeutic approaches for treating many illnesses [1]. With the advent of science-based ‘modern’ medicine, plant-based natural remedies for treating ailments came under intense scrutiny for their lack of scientific basis [1]. However, researchers kept seeking to identify the scientific basis of herbal remedies, medicinal plants, and functional foods. In recent decades, the emphasis on identifying therapeutic plant-based active principles led to significant advancements in the identification and use of natural compounds to treat various diseases (Figure 1) [2,3]. Much of the current knowledge of medicinal plants and their therapeutic properties derives from traditional Chinese or Indian medicine [3]. It is notable that an estimated 25–28% of modern medicines used by humanity, including those applied for the treatment of cancers, are directly or indirectly derivatives/compounds obtained from plants or other natural sources [3,4].

Cancers remain a major cause of death worldwide and significantly contribute to the social and economic burden. There remains an unmet need to develop cancer prevention strategies for those at risk and improve treatment strategies for the benefit of those already affected. Only a minor proportion of cancers are caused by hereditary or genetic predisposition. Cancers often develop over years or even decades, triggered by different processes involving DNA damage, epigenetic modifications, metabolic alterations, chronic inflammation, interactions between aberrant molecular pathways, inhibition of apoptosis, and cellular cross-talk with neighboring tissues. Interestingly, plant-based natural compounds can target one or more of these neoplasticity-triggering mechanisms and thus suppress the initiation, progression, metastatic spread, and relapse of cancers. 

*The Content of this Special Issue.* This Special Issue in *Biomolecules*, entitled “Plant-Derived Natural Compounds in the Management of Cancer: Significance and Challenges”, provides a broad and up-to-date overview of the significant aspects encompassing the research and developments in the use of plant-derived natural compounds in the treatment of various cancers.

Eighteen manuscripts are published in this Special Issue, including one feature paper from among eight published original research articles and one feature paper, as well as two editor’s choice articles, from among ten published review articles.

In their featured original article, Woo et al. [5] identified the ability of honokiol, a traditional Chinese-medicine-based natural biphenolic compound (extracted from *Magnolia* species), to target and sensitize cancer cells to undergo TRAIL-mediated apoptosis. Honokiol treatment in cancer cells correlated with the degradation and downregulation of anti-apoptotic survivin and c-FLIP. Interestingly, honokiol exposure led to the inhibition of STAMBPL1 (deubiquitinase), which, in turn, facilitated the ubiquitin–proteasome system-linked degradation of survivin and c-FLIP.

**Figure 1:**
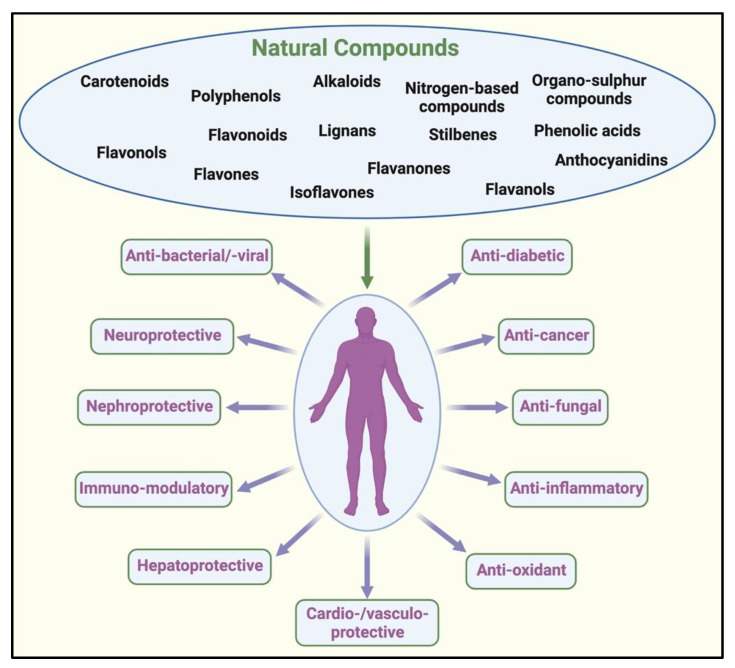
Pharmacological effects of natural compounds in various diseases. Naturally derived compounds have been used for their anti-oxidant, anti-diabetic, anti-cancer, anti-microbial, anti-inflammatory, immuno-modulatory, cardio-/vasculo-/hepato-/nephro-, and neuro-protective effects. Created with http://biorender.com/.

Takac et al. [6] investigated the effect of acridine chalcone 1C (AC1C) in human colorectal cancer cells. They observed that pro-oxidant properties of AC1C support the production of ROS and RNS, promoting mitochondrial dysfunction, DNA damage, and the activation of apoptosis in these cells via the activation of the MAPK-signaling mechanism. Using the anti-oxidant N-acetyl cysteine, the authors reversed the effect of AC1C, which further supported the anti-proliferative/pro-apoptotic effects of AC1C-induced oxidative stress in the colorectal cancer cells.

In most cancers, post-chemotherapy-induced leukopenia (CIL) significantly contributes to the higher mortality rates among patients. In their retrospective pilot study, Varughese et al. [7] indicated that a diet inclusive of green jackfruit flour, ‘Jackfruit365 (JF365)’, significantly reduced CIL in cancer patients who were also treated with pegfilgrastim. This study should pave the way for further in-depth investigations to identify the active component for this CIL-suppressing effect of JF365.

Abhinand et al. [8] studied the multiple anti-angiogenic targets of an herbal formulation, triphala churna (THL), prepared from dried fruits from three medicinal plants used in ancient Indian ayurvedic medicine. THL contains over a dozen different phytochemicals, each of which can pharmacologically inhibit tumor progression. However, with their approach of combining in silico (docking) and in vitro techniques, the team identified that punicalagin and chebulagic acid, among other components of THL, were key contributors inhibiting angiogenesis by targeting multiple components of the VEGF/VEGFR2 axis.

Clove is a well-recognized spice used in many cuisines all over the globe. Cloves are utilized to treat an upset stomach and as an expectorant. Clove oil is well known for its anesthetic effect, which helps to quickly relieve a toothache. Clove bud extracts (CBE) were used by Kello et al. [9] to showcase their ability to induce oxidative stress and DNA damage and activate apoptosis in human breast cancer cells. CBE treatment increased ROS- and RNS-related stress and activated the caspase-dependent apoptotic pathways while significantly modulating these cells’ Akt, p38MAPK, JNK, and ERK ½ pathways.

Isothiocyanates, which are abundant in cruciferous vegetables (Brassicaceae family such as broccoli, brussels sprouts, cabbage, and cauliflower), have been well studied for their chemopreventive chemotherapeutic effects in cancers. Treatment with Benzyl isothiocyanate (BITC), a degradation product of glucosinolates, found in edible plants of the Brassicaceae family, caused cell shrinkage and suppressed cell viability in gastric adenocarcinoma cells [10]. Han et al. [10] found that the BITC treatment-induced ROS production, and subsequent mitochondrial dysfunction, led to the mitochondria-mediated cytochrome-c release and caspase-dependent apoptosis. In another study, 6-(methylsulfinyl) hexyl isothiocyanate (6-MITC), a wasabi compound, inhibited the growth and viability of human leukemia cells by the concurrent induction of autophagy and mitotic arrest [11].

Nineteen different stilbenoids (phenolic compounds found in berries) were investigated by Treml et al. [12] to study their pro-/anti-oxidant properties in a cellular model of THP-1 macrophage-like cells. Their results show that the different stilbenoids can act as either pro-oxidants or anti-oxidants, and an in-depth study on how these characteristics can be used to combat various cancers is warranted.

Breast cancer remains the leading cause of cancer-related morbidity and mortality worldwide. Three of the review articles have focused on the potential anti-cancer effects of phytochemicals in breast cancers. In their featured review article, Varghese et al. [13] focused on the mechanism of tumor angiogenesis in light of the altered metabolism of tumor endothelial cells and how miRNAs influence this process in breast cancers. The authors looked through several miRNAs that modulated angiogenesis in breast cancer. They outlined several plant-derived natural compounds (cardamonin, resveratrol, silibinin, curcumin, metformin, genistein, triptolide luteolin, among others) that can alter several miRNA-dependent targets to block tumor angiogenesis and thus repress breast cancer growth and proliferation. In an editor’s choice review article from the same group, Samuel et al. [14] focused on the widely used anti-diabetic drug metformin as a cancer-preventive and chemotherapeutic agent in treating breast cancer. The article outlines the molecular mechanism of metformin action. It provides an in-depth discussion of the available cellular, pre-clinical, and clinical studies that have tested the anti-tumor potential of metformin as a potential anti-cancer/anti-tumor agent in breast cancer therapy. Abu Samaan et al. [15] reviewed the clinical effects of paclitaxel (PTX, a taxane compound first isolated from the Pacific yew tree), a commonly used chemotherapeutic drug, and provided mechanistic insights into its anti-cancer effect in different types of breast cancers. While discussing the novel advances in the application of PTX in breast cancers and the use of PTX in neoadjuvant therapy in combination with other anti-cancer drugs, the review also highlights its side effects, the development of resistance to PTX in breast tumors, and possible ways to overcome this treatment-induced resistance to PTX.

In a second editor’s choice article, Samec et al. [16] discuss the epigenetic post-translational histone modifications as the basis of antineoplastic effects of several phytochemicals in breast, prostate, and colorectal cancers. The authors provide the basis of histone modifications as molecular regulators of chromatin structure. They reviewed the effects of monotherapy and combination therapy using natural compounds on curbing breast, prostate, and colorectal cancers. Along the same theme of epigenetic modifications, Jasek et al. [17] reviewed the aberrant modifications in the function of DNA methyltransferases (DNMTs) as crucial triggers in the pathogenesis of human cancers. They looked at several pre-clinical and clinical studies that showcase the ability of phytochemicals and plant-based diets to target the epigenetic regulators and modulators of gene transcription and the activity of DNMTs and DNA methylation status in curbing tumor growth and progression.

Gastrointestinal (GI) cancer and its increasing incidence and rapid progression is the theme of Al-Ishaq et al.’s [18] article. The authors focus on several modifiable and non-modifiable risk factors for the increase in GI cancers and how several bioactive plant-derived secondary metabolites and diets rich in such phytochemicals reduce the incidence (chemopreventive effect) and progression (therapeutic effect) in cancers of the GI tract. They summarize several key molecular mechanisms/pathways (such as the PI3K/Akt, AMPK, mTOR, MAPK, NF- κB, Wnt/β-catenin pathways) that are modulated by natural compounds such as carotenoids, proanthocyanidins, isothiocyanates, and several other plant-metabolites to curb tumor growth and progression. 

Brain tumors (high-grade malignant gliomas, including glioblastoma and anaplastic astrocytoma) are among the most devastating and rapidly growing cancers. The chemotherapeutic effect of resveratrol (a polyphenolic component found in berries, nuts, grapes, and red wine) on malignant brain tumors is the focus of the review from Kiskova et al. [19]. The authors discuss in vitro and in vivo studies that pave the way for advanced clinical research in this area to test resveratrol and its efficacy in treating brain tumors.

Lichens are fascinating symbiotic organisms found in nature and capable of producing different phenolic compounds, including anthraquinones, xanthones, dibenzofurans, depsides, and depsidones. In their review article, Solárová et al. [20] discuss the molecular mechanisms responsible for the anti-neoplastic potential of several lichen-derived secondary metabolites. While Abotaleb et al. [21] reviewed the therapeutic potential of different plant-derived phenolic compounds in the treatment of cancer, Satheesh et al. [22] discuss the possibility that using vitamin C in combination with other conventional anti-cancer treatments can eradicate cancer stem cells (key contributors to therapeutic resistance, metastasis, and relapse).

*Are “Natural Substances” the Answer to More Efficient Anti-Cancer Therapy?* The articles published in this Special Issue prove that plant-based natural formulations are a vital source of chemopreventive and chemotherapeutic agents. Phytochemicals have a plethora of biological anti-cancer activities and could thus be a rational and practical approach to the effective treatment of cancers. While science and pharmaceutics have made significant advancements in chemically synthesized pharmacological anti-cancer agents and the early diagnosis and identification of cancers, the unfortunate truth is that modern medicine is desperately short of new and targeted therapeutic approaches. It takes years for a new drug to get through research and development and into clinical use, and this is accompanied by high costs [3]. The once sidelined, natural/plant-based remedies offer ways of relieving this crisis of drug development and harnessing naturally available active compounds to make cancer therapeutics more efficient, as well as more cost-effective and attainable to less privileged patients. 

Standard cancer treatment strategies (surgery and radiation) and routinely used chemotherapeutic drugs, in combination with natural bioactive compounds (Figure 2), could prove to be more efficient in treating cancers in terms of (1) a reduction in drug dosage, (2) alleviating side-effects, (3) overcoming drug resistance by re-sensitizing cancers to respond to drugs, (4) targeting cancer stem cells, and (5) curbing metastases and relapses in cancers [23,24].

With the advancements in genomics and understanding regarding the existence of genetic diversity between different patient populations, and even among individual patients, the world of modern medicine is fast embracing the concept of and need for preventive, personalized, precision medicine (3P medicine). In this scenario, when drugs tailored to treat patients on a case-by-case basis become important, turning to ‘natural sources’ of reliable drugs may help to ease the challenges. A serious clinical problem in cytotoxic anti-cancer therapies is the acquired resistance or insensitivity of cancer cells to conventional chemotherapeutics. The current research highlights the potential importance of phytochemicals in increasing chemotherapeutic agents’ sensitivity and/or efficacy against cancer [25]. Targeting specific molecular pathways by the use of plant nutraceuticals can improve therapeutic outcomes by increasing the sensitivity of cancer cells and reversing their resistance towards the currently applied therapeutic modalities, and thus represents an essential clinical approach to improving the clinical management of cancer. In this regard, testing conventional chemotherapies, in combination with phytopharmaceuticals, on patient-derived cancer cells, using progressive methods, can predict the patient responses and provide outputs for a personalized approach in an individual.

**Figure 2:**
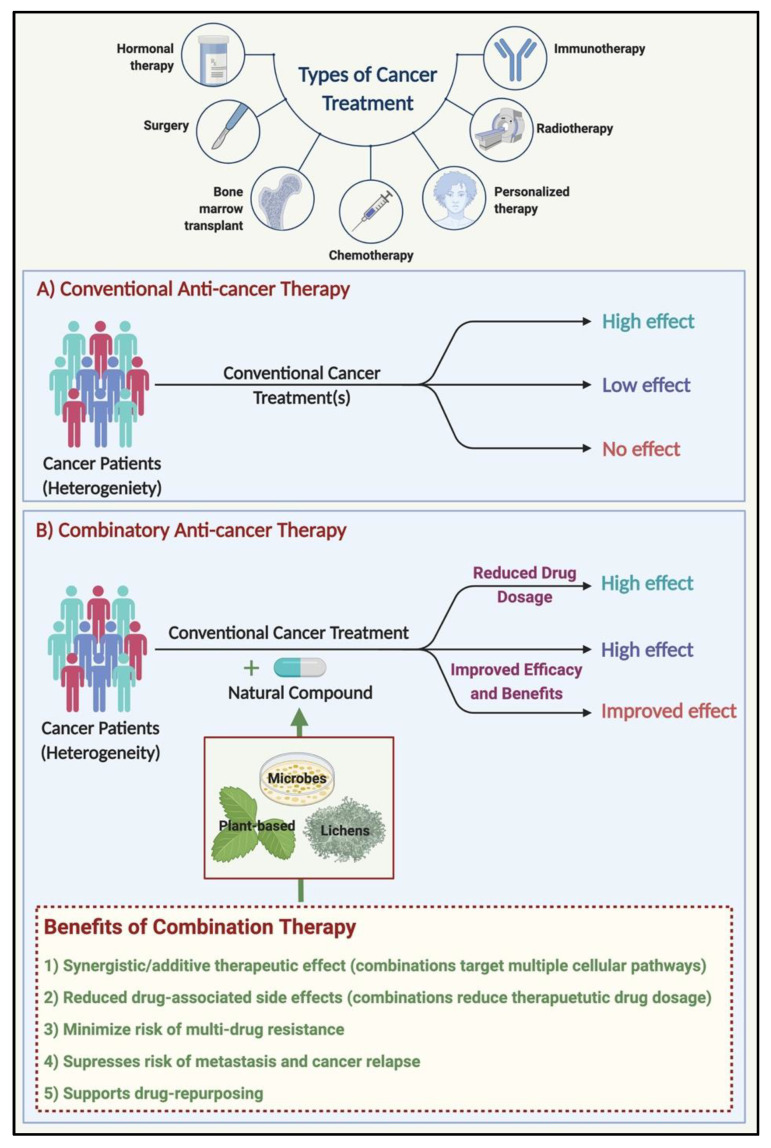
Benefits of combination therapy (using natural compounds in anti-cancer treatment). The conventional cancer treatments include surgical removal of the tumor, chemotherapy, and radiation therapy, in addition to the standard therapeutic procedures, depending on the type/sub-type of cancer. In a heterogeneous population of cancer patients, while conventional/standard treatment practices benefit some patients, in others, the treatment strategy may have little to no effect. In such a scenario, using a natural compound in combination with the conventional/standard treatment procedures may prove to be beneficial in several ways. Created with http://biorender.com/.

Although this Special Issue focuses on ‘plant-derived natural compounds in cancers’, we acknowledge that ‘natural sources’ for bioactive compounds with medicinal properties are found not just in terrestrial plants but throughout nature, and can help treat various other diseases, as well as cancer [26]. The marine environment, microbes (bacteria and fungi), slime molds, lichens, and unexpected sources of medicinal remedy, such as the saliva of the Gila monster (the compound found in the saliva, which turned out to be the basis for exenatide, a synthetic anti-diabetic drug), have yielded remarkable therapeutic agents for the treatment of numerous diseases [26]. Analgesics (painkillers), anti-biotics/anti-microbials, anti-malarials, drugs to treat metabolic and cardiovascular diseases as well as diseases of the nervous and digestive systems, and potential drugs to treat COVID-19, are just few examples of the many therapeutic benefits that humanity has received from from nature [27,28,29,30,31].

*Lessons to be Learned and Taught.* It is time that differences in opinions regarding the ‘better’ treatment option between practitioners of traditional and modern medicine be set aside, and the different groups work together for the greater good of humanity. On the one hand, practitioners of traditional natural medicine must be willing to share their knowledge. On the other hand, scientists, researchers, and clinicians must support and protect those who practice traditional/natural medicine and be willing to learn from them and their experiences. The knowledge gained from traditional medicine should go hand-in-hand with the pharmaceutical industry’s expertise in drug development [3]. The lack of international standardization when evaluating the composition, efficacy, safety, and quality of natural medicinal compounds in therapeutics must be overcome [3]. Coordination and collaboration is necessary between local, regional, national, and international drug regulatory agencies and the pharmaceutical industry to provide clear guidelines regarding the scientific data on the therapeutic effects of compounds derived from natural sources and the regulation/approval processes and manufacturing practices for new drugs [3].

While there is so much to thank nature for, there is so much more to gain from it. It is likely that we have barely scratched the surface of the medicines that nature has to offer us. Hence, there remains an imminent need to protect our planet from the threats of drastic climate change and unnecessary human interventions that erode and destroy natural resources and their molecular diversity, ultimately depriving humanity of potential sources of natural medicines. Let us play a part in protecting nature and, in turn, let nature protect us!

## References

[1] Yuan H., Ma Q., Ye L., Piao G. (2016). The Traditional Medicine and Modern Medicine from Natural Products. Molecules.

[2] Newman D.J., Cragg G.M. (2016). Natural Products as Sources of New Drugs from 1981 to 2014. J. Nat. Prod..

[3] Fridlender M., Kapulnik Y., Koltai H. (2015). Plant derived substances with anti-cancer activity: From folklore to practice. Front. Plant Sci..

[4] Chin Y.W., Balunas M.J., Chai H.B., Kinghorn A.D. (2006). Drug discovery from natural sources. AAPS J..

[5] Woo S.M., Seo S.U., Kubatka P., Min K.J., Kwon T.K. (2019). Honokiol Enhances TRAIL-Mediated Apoptosis through STAMBPL1-Induced Survivin and c-FLIP Degradation. Biomolecules.

[6] Takac P., Kello M., Vilkova M., Vaskova J., Michalkova R., Mojzisova G., Mojzis J. (2020). Antiproliferative Effect of Acridine Chalcone Is Mediated by Induction of Oxidative Stress. Biomolecules.

[7] Varughese T., Joseph J., Menon R. (2020). Efficacy of Jackfruit365™ Green Jackfruit Flour Fortified Diet on Pegfilgrastim to Prevent Chemotherapy-Induced Leukopenia, Irrespective of Tumor Type or Drugs Used-A Retrospective Study. Biomolecules.

[8] Abhinand C.S., Athira P.A., Soumya S.J., Sudhakaran P.R. (2020). Multiple Targets Directed Multiple Ligands: An In Silico and In Vitro Approach to Evaluating the Effect of Triphala on Angiogenesis. Biomolecules.

[9] Kello M., Takac P., Kubatka P., Kuruc T., Petrova K., Mojzis J. (2020). Oxidative Stress-Induced DNA Damage and Apoptosis in Clove Buds-Treated MCF-7 Cells. Biomolecules.

[10] Han K.W.W., Po W.W., Sohn U.D., Kim H.J. (2019). Benzyl Isothiocyanate Induces Apoptosis via Reactive Oxygen Species-Initiated Mitochondrial Dysfunction and DR4 and DR5 Death Receptor Activation in Gastric Adenocarcinoma Cells. Biomolecules.

[11] Wu K.M., Liao H.F., Chi C.W., Kou Y.R., Chen Y.J. (2019). Wasabi Compound 6-(Methylsulfinyl) Hexyl Isothiocyanate Induces Cell Death with Coexisting Mitotic Arrest and Autophagy in Human Chronic Myelogenous Leukemia K562 Cells. Biomolecules.

[12] Treml J., Leláková V., Šmejkal K., Paulíčková T., Labuda Š., Granica S., Havlík J., Jankovská D., Padrtová T., Hošek J. (2019). Antioxidant Activity of Selected Stilbenoid Derivatives in a Cellular Model System. Biomolecules.

[13] Varghese E., Liskova A., Kubatka P., Mathews Samuel S., Büsselberg D. (2020). Anti-Angiogenic Effects of Phytochemicals on miRNA Regulating Breast Cancer Progression. Biomolecules.

[14] Samuel S.M., Varghese E., Kubatka P., Triggle C.R., Büsselberg D. (2019). Metformin: The Answer to Cancer in a Flower? Current Knowledge and Future Prospects of Metformin as an Anti-Cancer Agent in Breast Cancer. Biomolecules.

[15] Abu Samaan T.M., Samec M., Liskova A., Kubatka P., Büsselberg D. (2019). Paclitaxel’s Mechanistic and Clinical Effects on Breast Cancer. Biomolecules.

[16] Samec M., Liskova A., Koklesova L., Mestanova V., Franekova M., Kassayova M., Bojkova B., Uramova S., Zubor P., Janikova K. (2019). Fluctuations of Histone Chemical Modifications in Breast, Prostate, and Colorectal Cancer: An Implication of Phytochemicals as Defenders of Chromatin Equilibrium. Biomolecules.

[17] Jasek K., Kubatka P., Samec M., Liskova A., Smejkal K., Vybohova D., Bugos O., Biskupska-Bodova K., Bielik T., Zubor P. (2019). DNA Methylation Status in Cancer Disease: Modulations by Plant-Derived Natural Compounds and Dietary Interventions. Biomolecules.

[18] Al-Ishaq R.K., Overy A.J., Büsselberg D. (2020). Phytochemicals and Gastrointestinal Cancer: Cellular Mechanisms and Effects to Change Cancer Progression. Biomolecules.

[19] Kiskova T., Kubatka P., Büsselberg D., Kassayova M. (2020). The Plant-Derived Compound Resveratrol in Brain Cancer: A Review. Biomolecules.

[20] Solárová Z., Liskova A., Samec M., Kubatka P., Büsselberg D., Solár P. (2020). Anticancer Potential of Lichens’ Secondary Metabolites. Biomolecules.

[21] Abotaleb M., Liskova A., Kubatka P., Büsselberg D. (2020). Therapeutic Potential of Plant Phenolic Acids in the Treatment of Cancer. Biomolecules.

[22] Satheesh N.J., Samuel S.M., Büsselberg D. (2020). Combination Therapy with Vitamin C Could Eradicate Cancer Stem Cells. Biomolecules.

[23] Dehelean C.A., Marcovici I., Soica C., Mioc M., Coricovac D., Iurciuc S., Cretu O.M., Pinzaru I. (2021). Plant-Derived Anticancer Compounds as New Perspectives in Drug Discovery and Alternative Therapy. Molecules.

[24] Samuel S.M., Varghese E., Koklesová L., Líšková A., Kubatka P., Büsselberg D. (2020). Counteracting Chemoresistance with Metformin in Breast Cancers: Targeting Cancer Stem Cells. Cancers.

[25] Liskova A., Samec M., Koklesova L., Brockmueller A., Zhai K., Abdellatif B., Siddiqui M., Biringer K., Kudela E., Pec M. (2021). Flavonoids as an effective sensitizer for anti-cancer therapy: Insights into multi-faceted mechanisms and applicability towards individualized patient profiles. EPMA J..

[26] Cragg G.M., Pezzuto J.M. (2016). Natural Products as a Vital Source for the Discovery of Cancer Chemotherapeutic and Chemopreventive Agents. Med. Princ. Pract..

[27] Varghese E., Samuel S.M., Liskova A., Kubatka P., Büsselberg D. (2021). Diabetes and coronavirus (SARS-CoV-2): Molecular mechanism of Metformin intervention and the scientific basis of drug repurposing. PLoS Pathog..

[28] Samuel S.M., Varghese E., Büsselberg D. (2021). Therapeutic Potential of Metformin in COVID-19: Reasoning for Its Protective Role. Trends Microbiol..

[29] Liskova A., Samec M., Koklesova L., Samuel S.M., Zhai K., Al-Ishaq R.K., Abotaleb M., Nosal V., Kajo K., Ashrafizadeh M. (2021). Flavonoids against the SARS-CoV-2 induced inflammatory storm. Biomed. Pharmacother..

[30] Kaul R., Paul P., Kumar S., Büsselberg D., Dwivedi V.D., Chaari A. (2021). Promising Antiviral Activities of Natural Flavonoids against SARS-CoV-2 Targets: Systematic Review. Int. J. Mol. Sci..

[31] Mathur S., Hoskins C. (2017). Drug development: Lessons from nature. Biomed. Rep..

